# Conclusion: What We Learned and Next Steps

**DOI:** 10.1177/1542305020988846

**Published:** 2021-04

**Authors:** Trace Haythorn

**Affiliations:** ACPE: The Standard for Spiritual Care and Education, Atlanta, USA

**Keywords:** Chaplain, COVID-19, spiritual care, global survey

## Abstract

In the previous articles, my colleagues have presented analyses of the survey data that have implications for professional preparation, practice and ongoing professional development. I will conclude this volume highlighting six areas that require further investigation, theoretical development and practical integration.

## Introduction

While there have certainly been other pandemics, COVID-19 has been unprecedented in the rapidity of its spread, mortality and long-term recovery implications. In this moment in history of 24/7/365 news coverage, the pervasiveness of the pandemic is relentless. Chaplains and spiritual care professionals have experienced COVID-19 in much the same way that the public has. For some, the intensity of the crisis has driven them to the point of exhaustion. For others, they have been sidelined by furloughs and redundancy actions by their institutions. Others have seen very little change, though no one responding to this survey went untouched by the virus and its ripple effects.

In the previous articles, my colleagues have presented analyses of the survey data that have implications for professional preparation, practice and ongoing professional development. I will conclude this volume highlighting six areas that require further investigation, theoretical development and practical integration.

### Standards

The survey gives clear evidence of the many ways chaplains and spiritual care professionals adapted their practices to the rapidly changing conditions that were implemented in efforts to contain the virus. Most of these efforts happened on the ground, at the grassroots level. COVID-19 made clear there is no coordinated mechanism that could provide guidance to frontline responders. Because the pandemic grew in waves, some chaplains in areas with rising incidences of cases learned from their colleagues whose “hot spots” had begun to cool about possible responses. Often, these responses did not address cultural and multi-faith competencies, relying instead on whatever might be available in the midst of the crisis.

Austyn Snowden raises important questions about the lack of coordination in the field. Is there a global standard for spiritual care? Should there be? If so, who decides and what governs such a standard? If not, what does that say about the role of spiritual care in overall wellness of patients? Of staff?

### Technological Adaptation

COVID-19 has clearly pushed chaplains into the age of mobile computing. Many had little time to learn new technologies and experimented with tools without much guidance. Rapid integration of new means of communicating and providing care to patients, staff and family. This survey only explored the use of such technologies; robust study is needed of best practices for these tools as they will likely become a part of whatever the “new normal” is for spiritual care for years to come. The use of technology also highlighted the sense of “absence presence,” a shift from the primary focus on physical and spiritual presence by spiritual care providers with those they serve to a presence through technological means in the interest of safety, thus providing care at a distance. While many may assume that these practices are substandard, what is lost and what is gained through such a strategy?

### Advocacy and Integration

The survey data makes clear that chaplains who experienced the greatest integration as a part of the healthcare team were most likely to be deemed “essential” in their context and were most likely to provide support for staff as well as patients (especially when patient care led to staff care). The constraints of the pandemic made it clear that chaplains and spiritual care professionals cannot assume that they are already integrated in the teams with whom they serve, with some being furloughed while others turned their focus to online support. We see a difference between those who were integrated and those who were not. Greater research is needed to identify effective strategies for cultivating these roles. In addition, educators must consider how professional advocacy for spiritual care can be included in the formation of chaplains.

### Community, Supervision, and Intervision/Collaboration

The survey demonstrates the vital role that community plays in the well-being of chaplains. “Community” includes interdisciplinary teams, colleagues, family, friends, professional association, and faith community. While spiritual care is often practiced in a one-on-one setting, the need for support, encouragement, and collegiality were very clear in survey responses. In many settings, while supervision was desired and/or needed, it was not available due to social distancing. In such contexts, intervision and interdisciplinary collaboration provided vital support. How are programs preparing chaplains to attend to these opportunities, i.e. intervision and interdisciplinary collaboration? How are current departments and divisions encouraging such practices? What opportunities are available to professionals to develop skills and strategies to build such networks of support? How do chaplains prevent forgetting to care for themselves while increasing staff care in times of crises (an issue addressed in the staff care and self-care articles)?

### Cultural/Religious Competencies

The survey data show deficits in cultural and religious competencies, especially when one considers the increasingly diverse contexts represented by the respondents, one in which care recipients draw upon less traditional/conventional religious forms. Data seems to indicate a shared value against proselytization; that said, respondents need more than referral networks to address the diverse spiritual needs of the people they serve. The data indicates less demand for chaplains to serve only those from their tradition. At the same time, we see that chaplains cope by drawing upon the inner resources linked to their own spirituality. Thus, it is also important information to enhance the knowledge of their spirituality, bring it in dialogue with frameworks, give them tools to work with it and to use it for their own survival. What pedagogical tools and practices are needed to effectively teach chaplains how they can be available to people across traditions without violating the integrity of either?

### Gaps/Next Steps

While the survey attempted to reach countries and ethnicities around the world, there are clear gaps. Much of the African continent and most of Asia are not represented due to our lack of networks there (note: the virus has not spread at the same level in much of Africa.) While some responses were received from Latin America, Europe, North America and Australia dominated the responses. In addition, what are the demographic diversities represented among chaplains and spiritual care professionals and the contexts they serve (e.g., socioeconomic, racial/ethnic, age, gender identity), and how do they inform/shape the practices of professional spiritual care?

## Conclusion

This research is clearly a start, but we see strong evidence of several things. First, chaplains are resilient and weary, challenged and creative, serving from wherever they safely can serve while also advancing the larger profession of spiritual care. Quite simply, chaplains on the whole have demonstrated that they are essential to the global response to COVID-19. Second, while there is great global diversity in healthcare, there are many consistent practices in professional spiritual care, including a rejection of proselytization, the importance of the spiritual resources and/or tradition of the chaplain, and the larger religious/spiritual context within which chaplains serve. Third, chaplains demonstrate a remarkable ability to support patients, staff and colleagues across traditions, positioning chaplaincy as an important force for a global, appreciative, religious leadership. Much more is needed to assess the efficacy of the items listed above. In addition, the gaps in the global responses highlight the challenge of research models that don’t privilege Western models of care or reinforce colonialist paradigms.

On behalf of all of the research team, a special word of thanks to Anne Vandenhoeck, Austyn Snowden, and Joost Verhoef for their vision and leadership in this effort.

